# Shortened Telomere Length Is Associated with Increased Risk of Cancer: A Meta-Analysis

**DOI:** 10.1371/journal.pone.0020466

**Published:** 2011-06-10

**Authors:** Hongxia Ma, Ziyuan Zhou, Sheng Wei, Zhensheng Liu, Karen A. Pooley, Alison M. Dunning, Ulrika Svenson, Göran Roos, H. Dean Hosgood, Min Shen, Qingyi Wei

**Affiliations:** 1 Department of Epidemiology, The University of Texas MD Anderson Cancer Center, Houston, Texas, United States of America; 2 Department of Epidemiology and Biostatistics, School of Public Health, Nanjing Medical University, Nanjing, China; 3 Cancer Research UK Genetic Epidemiology Unit, Department of Public Health and Primary Care, University of Cambridge, Strangeways Research Laboratory, Cambridge, United Kingdom; 4 Department of Oncology, University of Cambridge, Strangeways Research Laboratory, Cambridge, United Kingdom; 5 Department of Medical Biosciences/Pathology, Umeå University, Umeå, Sweden; 6 Division of Cancer Epidemiology and Genetics, National Cancer Institute, National Institutes of Health, Department of Health and Human Services, Bethesda, Maryland, United States of America; Ohio State University Medical Center, United States of America

## Abstract

**Background:**

Telomeres play a key role in the maintenance of chromosome integrity and stability, and telomere shortening is involved in initiation and progression of malignancies. A series of epidemiological studies have examined the association between shortened telomeres and risk of cancers, but the findings remain conflicting.

**Methods:**

A dataset composed of 11,255 cases and 13,101 controls from 21 publications was included in a meta-analysis to evaluate the association between overall cancer risk or cancer-specific risk and the relative telomere length. Heterogeneity among studies and their publication bias were further assessed by the χ^2^-based Q statistic test and Egger's test, respectively.

**Results:**

The results showed that shorter telomeres were significantly associated with cancer risk (OR = 1.35, 95% CI = 1.14–1.60), compared with longer telomeres. In the stratified analysis by tumor type, the association remained significant in subgroups of bladder cancer (OR = 1.84, 95% CI = 1.38–2.44), lung cancer (OR = 2.39, 95% CI = 1.18–4.88), smoking-related cancers (OR = 2.25, 95% CI = 1.83–2.78), cancers in the digestive system (OR = 1.69, 95% CI = 1.53–1.87) and the urogenital system (OR = 1.73, 95% CI = 1.12–2.67). Furthermore, the results also indicated that the association between the relative telomere length and overall cancer risk was statistically significant in studies of Caucasian subjects, Asian subjects, retrospective designs, hospital-based controls and smaller sample sizes. Funnel plot and Egger's test suggested that there was no publication bias in the current meta-analysis (*P* = 0.532).

**Conclusions:**

The results of this meta-analysis suggest that the presence of shortened telomeres may be a marker for susceptibility to human cancer, but single larger, well-design prospective studies are warranted to confirm these findings.

## Introduction

Telomeres, a series of tandem repeats of TTAGGG nucleotides, cap the ends of chromosomes in all eukaryotic cells [1] and maintain genomic stability by prohibiting fatal events, such as nucleolytic degradation, chromosomal end-to-end fusion and irregular recombination [2]. Human telomeres are approximately 10–15 kb in somatic cells and progressively shortened by ∼30 to 200 bp after each cycle of mitotic division, due to incomplete replication of linear DNA molecules and the absence of a mechanism for elongation of telomeres [3]. When the telomeres reach a critical length, Rb and p53 signaling pathways are triggered to initiate either cell senescence or apoptosis [4]. Thus, telomere length has been suggested as a “cellular mitotic clock” that defines the number of cell divisions and cellular life span [1], [5].

Several studies have documented correlations between shortened telomeres and multiple human diseases associated with age, such as Alzheimer's disease [6], myocardial infarction [7], vascular dementia [8], liver cirrhosis [9], atherosclerosis [10], ulcerative colitis [11] and premature aging syndromes [12]. Additionally, telomere shortening is involved in initiation and progression of malignancies in mouse models and functional studies [13], [14]. For example, short telomeres cause an increased risk of developing epithelial cancers by the formation of complex non-reciprocal translocations [15], [16], and telomeres in tumor cells and their precursor lesions are significantly shorter than that in surrounding non-tumor cells [17], [18].

Although evidence from functional studies and animal models support the hypothesis that telomere shortening contributes to tumor development, results from population studies remain conflicting rather than conclusive [19]–[44]. For instance, several case-control studies have investigated the association between telomere length in peripheral blood lymphocytes and breast cancer risk [21], [25], [29], [31], [35], [36], [38]; some showed that shorter telomeres were associated with increased risk of breast cancer [31], [38], while others indicated converse or insignificant associations [21], [25], [29], [35], [36]. These findings suggest that any of these single studies may have been underpowered to detect the association between telomere length and cancer risk because of their limited sample sizes. Furthermore, the underlying heterogeneity among different studies can be explored in a meta-analysis. Thus, we conducted a systematic meta-analysis on 21 relevant publications with 11,255 cases and 13,101 controls to estimate the overall cancer risk or cancer-specific risk associated with telomere length and to evaluate potential between-study heterogeneity of these published studies.

## Materials and Methods

### Search strategy and selection criteria

We used two electronic databases (MEDLINE and EMBASE) to identify all case-control studies published to date on an association between telomere length and cancer risk (last search update in November, 2010, using the search terms “telomere length”, “cancer” or “carcinoma”, and “risk”). Additional studies were identified by a hands-on search of references of original studies or reviews on this topic. Authors were also contacted directly, if crucial data were not reported in original papers. Studies included in the current meta-analysis had to meet the following criteria: written in English; case-control design; sufficient information needed to estimate odds ratios (ORs) and their 95% confidence intervals (CIs); independent from other studies to avoid double weighting in the estimates derived from the same study. In addition, investigations in subjects with cancer-prone disposition were excluded from the analysis.

### Data extraction

Two authors (HM and ZZ) independently extracted data and reached a consensus on all of the items. The following information was extracted from each report: the first author, year of publication, country of origin, ethnicity, cancer type, the number of cases and controls grouped by median or quartiles of relative telomere length (T/S ratio), study type, control source (population-based and hospital-based), DNA source, and measurement methods for telomere length. For studies including subjects of different racial descent, data were extracted separately for each ethnic group (categorized as Caucasian, Asian or others). When a study did not state what ethnic groups were included or if it was impossible to separate participants according to the data presented, the sample was termed as ‘other populations’. Furthermore, references involved in different ethnic groups, different types of cancer and different institutions were divided into multiple study samples for subgroup analyses.

### Quantitative data synthesis

The number of cases and controls grouped by the median of the relative telomere length (T/S ratio) was collected from each study to evaluate the risk of cancers (ORs and 95% CI). For each study, a median value of the relative telomere length (T/S ratio) in controls was considered as a cut-point dividing all subjects into two groups: the longer telomere group and the shorter telomere group. The association between the relative telomere length (T/S ratio) and cancer risk was examined by ORs and 95% CIs with the group of longer telomeres as the reference. The stratification analyses were also conducted by cancer type (if one cancer type was investigated in less than three studies, it would be merged into the ‘other cancers’ group), study type (retrospective and prospective), ethnicity (Caucasian, Asian or others), control source (hospital-based and population-based) and sample size (<500, 500–1000 and >1000). Smoking-related cancers were defined as those of the lung, bladder, head and neck, kidney and pancreas; and cancers of the digestive system included those of the stomach, esophagus and colon. Additionally, cancers arising from the bladder, kidney and prostate sites were considered cancers of the urogenital system.

The χ^2^-based Q test was performed to assess between-study heterogeneity and considered significant if *P*<0.05 [45]. Heterogeneity was also quantified with the *I* ^2^ statistic, a value that indicates what proportion of the total variation across studies is beyond chance, where 0% indicates no observed heterogeneity and larger values show increasing heterogeneity [46]. The fixed-effects model and the random-effects model, based on the Mantel-Haenszel method [47] and the DerSimonian and Laird method [48], respectively, were used to combine values from different studies. When *P* value of the heterogeneity test was ≥0.05, the fixed-effects model was used, which assumes the same homogeneity of effect size across all studies; otherwise, the random-effects model was more appropriate, which tends to provide wider confidence intervals, when the results of the constituent studies differ among themselves. To evaluate the effect of individual studies on the overall risk of cancers, sensitivity analyses were performed by excluding each study individually and recalculating the ORs and 95% CI. Furthermore, a sensitivity analysis was also performed each by excluding three studies whose matching information was unavailable [21], [25], [35], two studies whose DNA were not from blood [20], [34], and three studies that did not use quantitative PCR to test relative telomere length(T/S ratio) [19], [22], [36]. The inverted funnel plots and Egger's test (linear regression analysis) were used to investigate publication bias [49]. All analysis was conducted by using Review Manage (v.5.0) and Stata 10.0. All *P* values were two-sided.

## Results

### Characteristics of Studies

**Figure 1:**
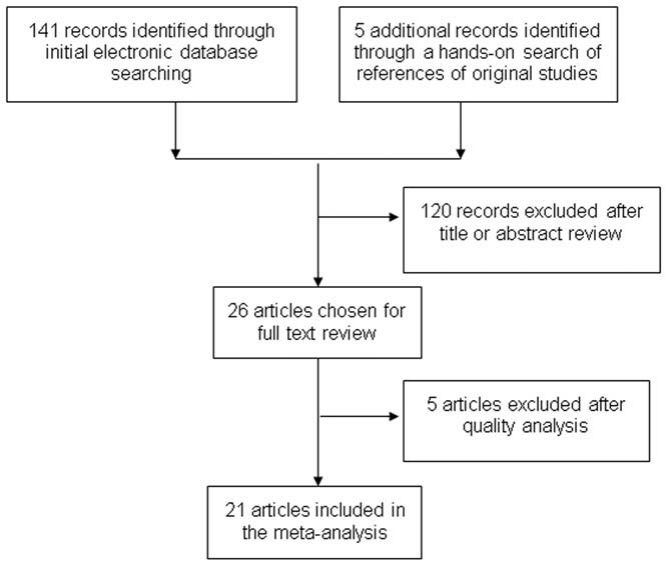
Flow chart for the process of selecting the final 21 publications.

As shown in **Fig. 1**, a total of 146 published records were retrieved by using the key words mentioned earlier in the Methods, of which 26 examined the association between telomere length and cancer risk. Among those 26 publications, five were excluded either because they did not provide available data to extract the ORs and 95% CI [40], [41], [43], [44] or the subjects were of cancer-prone predisposition [42]. The remaining 21 publications of case-control studies contained 29 studies (Wu's and Pooley's studies had datasets of four different cancers and McGrath's and Zheng's studies had datasets of two different sources) [19], [23], [36], [38]. The essential information, including first author, year of publication, country, ethnicity, cancer type, numbers of cases and controls, study type, control source and DNA source for all studies are listed in **Table 1**. Our meta-analysis included nine breast cancer studies [21], [29], [31], [35], [36], [38], four bladder studies [19], [20], [23], three lung cancer studies [19], [24], [34], two renal cancer studies [19], [22], two gastric cancers [27], [30], two colorectal cancers [38] and seven studies of other cancers [19], [26], [28], [32], [33], [37] (**Table 1**). Because some controls in one publication [19] were shared by different cancers, it was defined as four studies (head and neck cancer, bladder cancer, lung cancer and renal cell carcinoma) in the analysis stratified by tumor type but defined as one study in the overall analysis and stratification analysis by ethnicity, study type, control source and sample size. Overall, 15 studies used Caucasians, three used Asians, and eight used other ethnic groups; in addition, nine studies were prospective and seventeen were retrospective; 18 studies were population-based, seven were hospital-based, and one was family-based [21]. Most of studies provided matching information by age and/or other variables except for three studies [21], [25], [35]. The quantitative PCR was the most frequently used method to measure the relative telomere length (T/S ratio), while three studies used other assays including southern blot telomere restriction fragment (TRF) and quantitative fluorescence in situ hybridization-based approaches (Q-FISH) [19], [22], [36]. Additionally, the blood was the most common source of DNA, although other sources were also applied, such as buccal cells and sputum [20], [34].

**Table 1:** Characteristics of studies included in the meta-analysis.

Author	Year	Country	Ethnicity	Cancer type	cases/controls	Study type	Control source	DNA source	Measurement methods
Wu [19]	2003	USA	Caucasian	Head and neck cancer	92/92	Retrospective	Hospital-based	Lymphocytes	Southern Blot Analysis
Wu [19] a	2003	USA	Caucasian	Bladder cancer	135/135	Retrospective	Hospital-based	Lymphocytes	Q-FISHLSC
Wu [19] a	2003	USA	Caucasian	Lung cancer	54/54	Retrospective	Hospital-based	Lymphocytes	Q-FISHLSC
Wu [19] a	2003	USA	Caucasian	Renal cell carcinoma	32/32	Retrospective	Hospital-based	Lymphocytes	Q-FISHLSC
Broberg [20]	2005	Sweden	Caucasian	Bladder cancer	63/93	Retrospective	Population-based	Buccal cells	Quantitative PCR
Shen [21]	2007	USA	Mixed	Breast cancer	283/347	Retrospective	Family-based	White blood cells	Quantitative PCR
Shao [22]	2007	USA	Mixed	Renal Cancer	65/65	Retrospective	Hospital-based	Lymphocytes	Q-FISHLSC
McGrath [23]	2007	USA	Not defined	Bladder cancer (NHS)	61/67	Prospective	Population-based	Buffy coat	Quantitative PCR
McGrath [23]	2007	USA	Not defined	Bladder cancer (HPFS)	123/125	Prospective	Population-based	Buffy coat	Quantitative PCR
Jang [24]	2008	Korea	Asian	Lung cancer	243/243	Retrospective	Hospital-based	Whole blood	Quantitative PCR
Svenson [25]	2008	Sweden	European	Breast cancer	265/446	Retrospective	Population-based	Buffy coat, granulocyte	Quantitative PCR
Mirabello [26]	2009	USA	Caucasian	Prostate cancer	612/1049	Prospective	Population-based	Buffy coat	Quantitative PCR
Liu [27]	2009	China	Asian	Gastric cancer	396/378	Retrospective	Hospital-based	Whole blood	Quantitative PCR
Xing [28]	2009	USA	Caucasian	Esophageal cancer	94/92	Retrospective	Hospital-based	Whole blood	Quantitative PCR
De Vivo [29]	2009	USA	Caucasian	Breast cancer	896/917	Prospective	Population-based	Lymphocytes	Quantitative PCR
Hou [30]	2009	Poland	Caucasian	Gastric cancer	300/416	Retrospective	Population-based	Lymphocytes	Quantitative PCR
Shen [31]	2009	USA	Mixed	Breast cancer	1026/1070	Retrospective	Population-based	Mononuclear cells	Quantitative PCR
Lan [32]	2009	Finland	Caucasian	Non-Hodgkin Lymphoma	107/107	Prospective	Population-based	Whole blood	Quantitative PCR
Han [33]	2009	USA	Caucasian	Skin cancer	740/801	Prospective	Population-based	Buffy coat	Quantitative PCR
Hosgood [34]	2009	China	Asian	Lung cancer	109/97	Retrospective	Population-based	Sputum	Quantitative PCR
Gramatges [35]	2010	USA	Mixed	Breast cancer	102/50	Retrospective	Population-based	Whole blood	Quantitative PCR
Zheng [36]	2010	USA	Mixed	Breast cancer (RPC1)	152/176	Retrospective	Hospital-based	Buffy coat	Quantitative PCR
Zheng [36]	2010	USA	Mixed	Breast cancer (LCCC)	140/159	Retrospective	Hospital-based	Buffy coat	Q-FISHLSC
Mirabello [37]	2010	Poland	Caucasian	Ovarian cancer	98/100	Retrospective	Population-based	Buffy coat	Quantitative PCR
Pooley [38]	2010	UK	Caucasian	Breast cancer (SEARCH)	2243/2181	Retrospective	Population-based	Blood	Quantitative PCR
Pooley [38]	2010	UK	Caucasian	Breast cancer (EPIC)	199/420	Prospective	Population-based	Blood	Quantitative PCR
Pooley [38]	2010	UK	Caucasian	Colorectal cancer (SEARCH)	2161/2249	Retrospective	Population-based	Blood	Quantitative PCR
Pooley [38]	2010	UK	Caucasian	Colorectal cancer (EPIC)	185/406	Prospective	Population-based	Blood	Quantitative PCR
Prescott [39]	2010	USA	Caucasian	Endometrial cancer	279/791	Prospective	Population-based	Blood	Quantitative PCR

a Some controls were shared. PCR, polymerase chain reaction; Q-FISHLSC, quantitative fluorescence in situ hybridization-based approaches.

### Meta-analysis results

**Figure 2:**
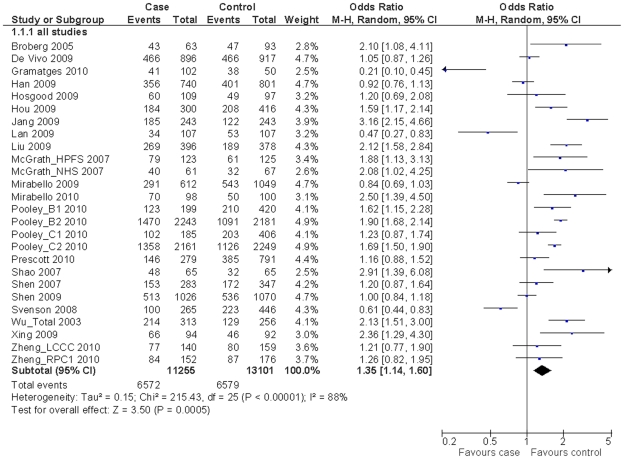
Odds ratios (ORs) and 95% confidence intervals (CIs) for overall cancer risk associated with relative telomere length (shorter *vs.* longer, grouped by the median of telomere length ratio). ^a^ Some controls were shared in the study by Wu *et al* (2003) that included a total of 313 cases and 256 controls.

**Figure 3:**
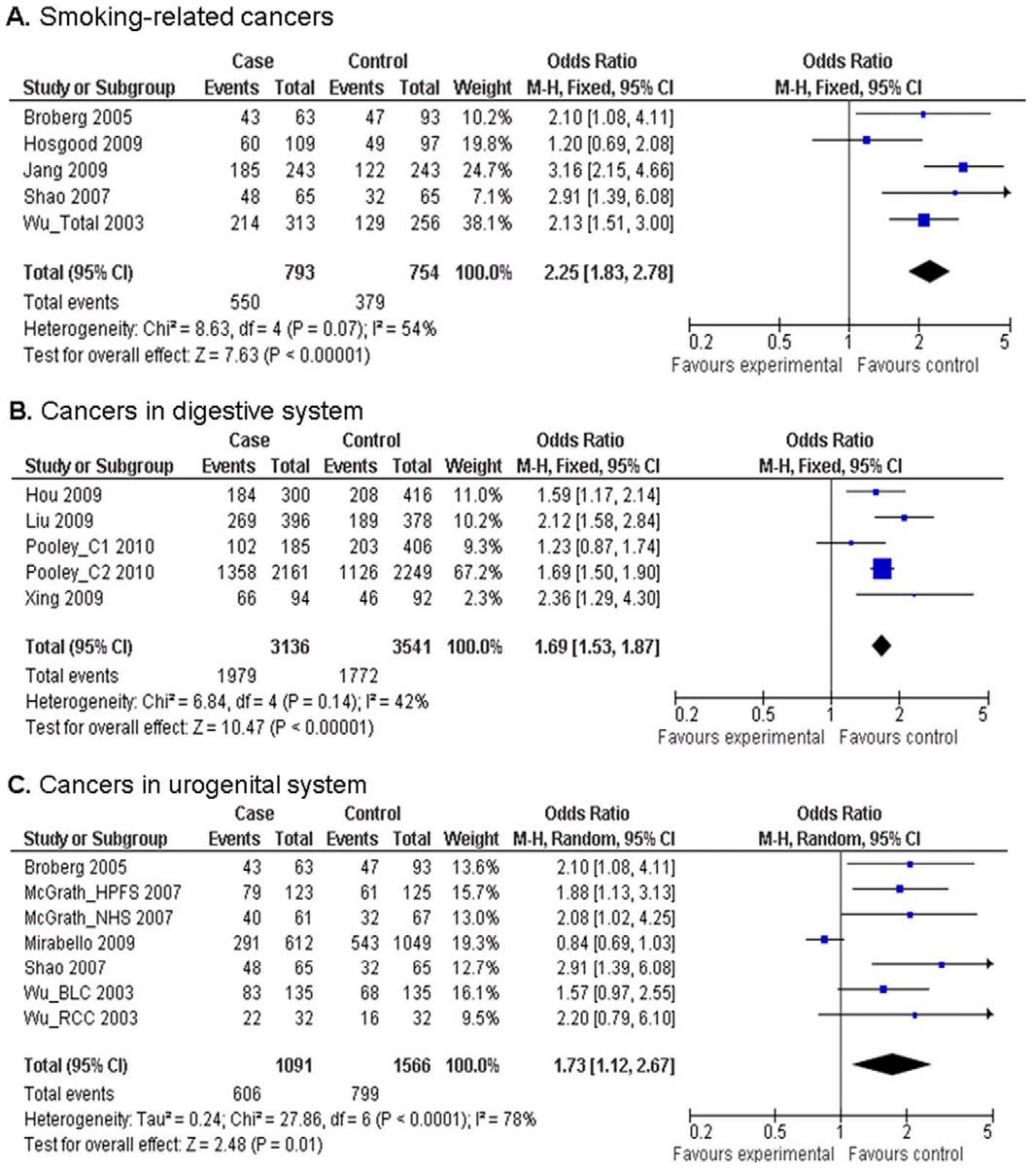
Odds ratios (ORs) and 95% confidence intervals (CIs) for risk of different cancers associated with relative telomere length (shorter *vs.* longer, grouped by median value of telomere length ratio). (**A**) Smoking-related cancers; (**B**) Cancers in the digestive system; (**C**) Cancers in the urogenital system.

We obtained the telomere genotyping data from 21 publications consisting of 11,255 cases and 13,101 controls. When all eligible studies were pooled into the meta-analysis, we found that shorter telomeres were significantly associated with the overall cancer risk (OR = 1.35, 95% CI = 1.14–1.60, *P*<0.001 for heterogeneity test, *I* ^2^ = 88%; **Fig. 2**). In the stratified analysis by tumor type (**Table 2**), the comparisons showed that individuals with shorter telomeres had an increased risk of bladder cancer (OR = 1.84, 95% CI = 1.38–2.44, *P* = 0.88 for heterogeneity test, *I* ^2^ = 0%) and lung cancer (OR = 2.39, 95% CI = 1.18–4.88, *P* = 0.009 for heterogeneity test, *I* ^2^ = 79%); but not breast cancer (OR = 1.04, 95% CI = 0.77–1.40, *P*<0.001 for heterogeneity test, *I* ^2^ = 92%). We also found the association between the relative telomere length and overall cancer risk was statistically significant in studies of Caucasian subjects (OR = 1.30, 95% CI = 1.06–1.61, *P*<0.001 for heterogeneity test, *I* ^2^ = 90%), Asian subjects (OR = 2.08, 95% CI = 1.31–3.30, *P*<0.001 for heterogeneity test, *I* ^2^ = 75%), retrospective design (OR = 1.44, 95% CI = 1.13–1.84, *P*<0.001 for heterogeneity test, *I* ^2^ = 86%), hospital-based controls (OR = 2.01, 95% CI = 1.54–2.62, *P* = 0.01 for heterogeneity test, *I* ^2^ = 62%), and sample sizes less than 500 (OR = 1.51, 95% CI = 1.06–2.16, *P*<0.001 for heterogeneity test, *I* ^2^ = 83%). Furthermore, when cancers were grouped into site-specific types (**Fig. 3**), the results showed that the association remained significant for smoking-related cancers (OR = 2.25, 95% CI = 1.83–2.78, *P* = 0.07 for heterogeneity test, *I* ^2^ = 54%), cancers in the digestive system (OR = 1.69, 95% CI = 1.53–1.87, *P* = 0.14 for heterogeneity test, *I* ^2^ = 42%) and in the urogenital system (OR = 1.73, 95% CI = 1.12–2.67, *P*<0.001 for heterogeneity test, *I* ^2^ = 78%).

**Table 2:** Associations between relative telomere length and cancer risk stratified by selected factors.

Variables	No of studies a	Sample	Shorter *vs.* longer		*P* for Heterogeneity
		Case/control	OR(95%CI)^b^	OR(95%CI)^c^	
**All**	26	11,255/13,101	1.35 (1.14–1.60)	1.37 (1.30–1.44)	<0.00001
**Tumor type**					
Breast cancer	9	5,306/5,766	1.04 (0.77–1.40)	1.29 (1.20–1.40)	<0.00001
Bladder cancer	4	382/420	1.83 (1.38–2.44)	1.84 (1.38–2.44)	0.88
Lung cancer	3	406/394	2.39 (1.18–4.88)	2.44 (1.82–3.27)	0.009
Other	13	5,161/6,578	1.47 (1.15–1.87)	1.37 (1.27–1.47)	<0.00001
**Ethnicity**					
Caucasian	15	8,555/10,324	1.30 (1.06–1.61)	1.38 (1.30–1.46)	<0.00001
Asian	3	748/718	2.08 (1.31–3.30)	2.20 (1.78–2.72)	<0.00001
Other	8	1,952/2,059	1.21 (0.87–1.70)	1.11 (0.98–1.26)	<0.00001
**Study type**					
Prospective	9	7,222/8,287	1.21 (0.93–1.57)	1.39 (1.30–1.48)	<0.00001
Retrospective	17	4,033/4,814	1.44 (1.13–1.84)	1.33 (1.22–1.45)	<0.00001
**Control Source**					
Hospital	7	1,403/1,369	2.01 (1.54–2.62)	2.03 (1.74–2.36)	0.01
Population	18	9,569/11,385	1.18 (0.96–1.43)	1.30 (1.23–1.38)	<0.00001
**Sample size**					
<500	13	1,670/1,630	1.51 (1.06–2.16)	1.61 (1.40–1.85)	<0.00001
500–1000	6	1,628/2,413	1.30 (0.91–1.86)	1.31 (1.15–1.49)	<0.00001
>1000	7	7,957/9,058	1.18 (0.91–1.53)	1.34 (1.26–1.42)	<0.00001

a Some controls in the publication by Wu (2003) *et al* were shared by different cancers; therefore, it was defined as four studies (head and neck cancer, bladder cancer, lung cancer and renal cell carcinoma) in the analysis stratified by tumor type, but defined as one study in the analysis stratified by study type, ethnicity and source of controls. In addition, the publication by Shen (2007) *et al* was family-based and excluded from the analysis for source of controls.

b Random effects model.

c Fixed effects model.

### Heterogeneity and sensitivity analyses

Substantial heterogeneity was observed among all studies for the relative telomere length and cancer risk (χ^2^ = 215.43, *P*<0.001, **Fig. 2**). Therefore, we evaluated the source of heterogeneity by tumor type, ethnicity, control source, study type and sample size, and we found that tumor type and control source did contribute to substantial heterogeneity (χ^2^ = 9.33, *P* = 0.025 for tumor type and χ^2^ = 9.88, *P* = 0.002 for control source, respectively) but not from ethnicity (χ^2^ = 3.90, *P* = 0.143), study type (χ^2^ = 0.91, *P* = 0.340) and sample size (χ^2^ = 1.21, *P* = 0.547). The leave-one-out sensitivity analysis indicated that no single study changed the pooled ORs qualitatively (data not shown). Furthermore, the sensitivity analysis without three studies whose matching information was unavailable [21], [25], [35], two studies whose DNA were not from blood [20], [34], or three studies without use of quantitative PCR to test relative telomere length (T/S ratio) [19], [22], [36] did not alter the results of the meta-analysis (OR = 1.48, 95% CI = 1.26–1.74, *P*<0.001 for heterogeneity test, *I* ^2^ = 87%; OR = 1.34, 95% CI = 1.12–1.59, *P*<0.001 for heterogeneity test, *I* ^2^ = 89%; and OR = 1.30, 95% CI = 1.08–1.55, *P*<0.001 for heterogeneity test, *I* ^2^ = 89%; respectively).

### Publication bias

**Figure 4:**
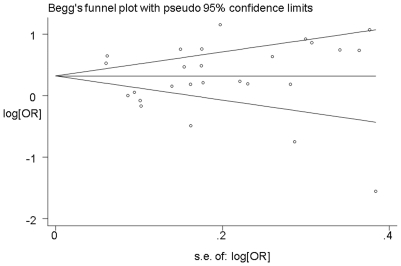
Funnel plot analysis to detect publication bias. Each point represents an independent study for the indicated association.

As shown in **Fig. 4**, the shapes of the funnel plots seemed symmetrical, and Egger's test suggested that there was no publication bias in the current meta-analysis (*P* = 0.532). These indicated that bias from publications might not have a significant influence on the results of our meta-analysis on the association between telomere length and cancer risk.

## Discussion

In this meta-analysis of 11,255 cancer cases and 13,101 controls from 21 independent publications, we found that shorter telomeres were significantly associated with risk of cancer, especially cancers of the bladder and lung, smoking-related, the digestive system and the urogenital system. Furthermore, the stratification analysis also showed that the association was more prominent in studies of Caucasian subjects, Asian subjects, retrospective design, hospital-based controls, and smaller sample sizes.

Studies have showed that telomeres are critical for maintaining genomic integrity and that telomere dysfunction or shortening is an early, common genetic alteration acquired in the multistep process of malignant transformation [12], [50]. In addition, telomere dysfunction has been found to be associated with decreased DNA repair capacity and complex cytogenetic abnormalities [51]. Both of animal studies and clinical observations have shown that shorter telomeres were associated with increased risk of cancers, such as epithelial cancers [52], [53], [54]. However, telomere shortening might play conflicting roles in cancer development. For example, the progressive loss of telomeric repeats with each cell division can induce replicative senescence and limit the proliferative potential of a cell, thus functioning as a tumor suppressor [12], [55]. But, once telomeres reach a critical length, it will result in chromosome break, causing genome instability and enhancing potential for malignant transformation via fusion-bridge-breakage cycles [56]. In this meta-analysis, we found that shorter telomeres were significantly associated with cancer risk, supporting the hypothesis that excessive telomere shortening may play a role in accelerating tumor onset and progression.

Although this meta-analysis showed significant associations between shorter telomeres and overall cancer risk, some results from stratification analysis remind us of drawing the conclusion with caution. The stratification analysis by tumor type showed that the association between shorter telomeres and cancer risk was significant in bladder cancer, lung cancer, smoking-related cancers, and cancers in the digestive system and in the urogenital system, but not in breast cancer. Because our heterogeneity analysis also showed that tumor type did contribute to substantial heterogeneity, these inconsistent results by cancer types may involve different carcinogenic mechanisms conferred by specific telomeres in specific cancer types. Different biological pathways (such as metabolisms of hormone, tobacco carcinogens and repair of DNA damage) could interact with telomere length, resulting in different efforts on cancer susceptibility. For example, several studies found that the effect of shortened telomeres on breast cancer risk was significant for certain subgroups, such as premenopausal women and women with a poor antioxidative capacity [31] but not for the overall study population [31], [36]or postmenopausal women[29]. The possible explanation may be that the difference in hormones, particularly estrogen, may affect telomere dynamics through its antioxidant attributes and its ability to stimulate telomerase, which can elongate telomere ends [57]. In addition, it has been reported that short telomeres on specific chromosome arms may be more important for cancer risk than the overall telomere length in a cell, and chromosome arms with the shortest telomeres were more often found in the telomere fusions, leading to chromosome instability [58], [59]. For breast cancer, frequent chromosomal abnormalities mainly occur on certain chromosome arms, such as gains of 1q, 8q, 17q, and 20q, and losses of 8p, 9p, 16q, and 17p [60], [61], [62]. Thus, different associations between overall telomere length and risk of different cancers may due to the confounding effect of large number of “irrelevant” telomeres in the measurement.

Furthermore, the results from stratification analysis by other factors, including study type, control source and sample size, indicated that the association was more prominent in studies with retrospective designs, hospital-based controls and smaller sample sizes. However, these studies suffer from several major drawbacks, such as information bias, selection bias and lower statistical power, which may have a substantial influence on the results of studies per se and thus on our meta-analysis as well. Specially, the difference between studies with retrospective designs (random effects model: OR = 1.44, 95% CI = 1.13–1.84; heterogeneity *P*<0.0001) and prospective designs (random effects model: OR = 1.21, 95% CI = 0.93–1.57; heterogeneity *P*<0.0001) suggests possible biases in those studies with retrospective designs. The majority of published studies on telomere length and cancer risk were retrospective case-control studies in which DNA samples from the cases were collected after cancer diagnosis. This could potentially result in reverse causation bias, where changes in surrogate tissue telomere length may be a consequence of the cancer rather than a cause. Recently, a study by Nordfjäll *et al* [63] evaluated the blood telomere length in 959 individuals at baseline and after 10 years of follows-up, and they found no differences in telomere length (at baseline or at follow up) between controls and those who later were diagnosed with cancer, which may challenge the hypothesis that individual telomere length can predict later tumor development. Therefore, the findings of an association between shorter telomeres and cancer risk in this meta-analysis still require further replication in single large prospective studies that avoid or carefully address potential biases.

### Limitations

Some other issues in this meta-analysis also need to be addressed. Firstly, several variables may affect the length of telomeres, such as age, sex, obesity, smoking, oxidative stress and chronic inflammation [12], [64], [65], [66]. However, the results of this meta-analysis were based on unadjusted estimates, because either ORs derived from different studies were not adjusted by the same potential confounders or only the number of cases and controls was provided without the detailed information of other variables. In fact, we did try to calculate the summary ORs using adjusted ORs available from only nine original papers [21], [24], [26], [27], [28], [29], [31], [36], [39], and we found that there were no substantial changes in the pooled, adjusted ORs (OR = 1.41, 95% CI = 1.10–1.82, *P*<0.001 for heterogeneity test, *I* ^2^ = 82.9%). Further, there is some evidence that treatment status (chemotherapy or radiation) can alter telomere length [67], [68], and we cannot rule out the possibility of such an effect because of unavailable information about the disease treatment status from the studies used in the analysis. A more precise analysis should be conducted, if individual data were available, allowing for the adjustment by some co-variants and excluding those patients who had been treated. Secondly, various methods were used to measure the relative telomere length in those studies used in our meta-analysis, including southern blot, Q-FISH and Q-PCR assays, which made it difficult to directly compare or pool data from different studies. Thirdly, the association between telomere length and cancer risk may be affected by the types of surrogate tissues. In studies included in this meta-analysis, DNA from multiple sources was used, including blood, buccal cells and sputum (**Table 1**). Although the majority of the inter-individual variation in telomere length may be genetically determined [69], and cells with different origins show a good intra-individual correlation for telomere length in healthy subjects and case subjects [70], [71], it may be disputable for the use of hematopoietic cells to be a proxy of average individual telomere length, because the variation in telomere length has been observed within leukocyte subsets but not others [72]. Therefore, consistent measurement methods and use of the surrogate tissues are warranted in further studies on telomere length, which may provide comparable data from different studies.

### Conclusions

Our meta-analysis provided statistical evidence for an association between shorter telomere length and risk of human cancer, particularly for bladder cancer, lung cancer, smoking-related cancers, and cancers in the digestive system and in the urogenital system. However, due to the limitations of original studies included in the meta-analyses, larger, well-designed prospective studies are needed to confirm these findings, which may help unravel the underlying mechanisms of telomere shortening in cancer development and progression.
